# Comparison of porous and nano zinc oxide for replacing high-dose dietary regular zinc oxide in weaning piglets

**DOI:** 10.1371/journal.pone.0182550

**Published:** 2017-08-08

**Authors:** Lina Long, Jiashun Chen, Yonggang Zhang, Xiao Liang, Hengjia Ni, Bin Zhang, Yulong Yin

**Affiliations:** 1 College of Animal Science and Technology, Hunan Agriculture University, Hunan, P. R. China; 2 Key Laboratory for Agro-Ecological Processes in Subtropical Region, Hunan Research Center of Livestock and Poultry Sciences, South Central Experimental Station of Animal Nutrition and Feed Science in the Ministry of Agriculture, Institute of Subtropical Agriculture, The Chinese Academy of Sciences, Hunan, P. R. China; 3 Animine Co., Ltd, Sillingy, France; 4 College of Veterinary Medicine, Qingdao Agricultural University, Qingdao, Shandong, P. R. China; Institute of Materials Science, GERMANY

## Abstract

The aim of this study was to compare the effect of dietary supplementation with low dose of porous and nano zinc oxide (ZnO) on weaning piglets, and to evaluate the possibility of using them as an alternative to high dose of regular ZnO. Piglets were randomly allocated into four treatment groups fed with four diets: (1) basal diet (NC), (2) NC+ 3000 mg/kg ZnO (PC), (3) NC + 500 mg/kg porous ZnO (HiZ) and (4) NC + 500 mg/kg nano ZnO (ZNP). The result showed that piglets in HiZ group had less diarrhea than ZNP group (*P* < 0.05). Besides, there was no significant difference between PC, HiZ and ZNP groups in terms of serum malondialdeyhde (MDA) concentration and glutathione peroxidase (GSH-Px) activity (*P* > 0.05). Analysis of trace metal elements revealed that piglets fed with high dose of regular ZnO had the highest Zn level in kidney (*P* < 0.05), which may induce kidney stone formation. Additionally, a decrease in ileal crypt depth was observed in PC, HiZ and ZNP group, suggesting an effective protection against intestinal injury. Results of mRNA analysis in intestine showed that ZNP supplementation had better effects on up-regulated trefoil factor 3 (TFF3) and nuclear factor erythroid 2-related factor 2 (Nrf2) levels in duodenum and jejunum than HiZ did (*P* < 0.05), implying that nano ZnO may possess higher anti-inflammatory capacity than porous ZnO. In conclusion, dietary supplementation with low dose of porous and nano ZnO had similar (even better) effect on improving growth performance and intestinal morphology, reducing diarrhea and intestinal inflammatory as high dose of regular ZnO in weaning piglets. Compared with nano ZnO, porous ZnO had better performance on reducing diarrhea but less effect on up-regulation of intestinal TFF3 and Nrf2.

## Introduction

Zinc oxide (ZnO) is a multifunctional material because of its diverse properties [1]. It plays an important role in a very wide range of applications, ranging from ceramics to tyres [2], from pharmaceuticals to agriculture [3, 4], and from chemicals to dye[5, 6]. Due to its antibacterial, disinfecting and drying properties, ZnO is widely used in the production of medicines for epilepsy [7], diarrhea [8] and wound healing[9]. At the present time, the advent of nanotechnology has brought great opportunities for the development of ZnO in many areas. ZnO in nanoscale has shown potential applications in food preservation [3], cancer control [10, 11] and anti-viral treatment [12].

In swine production, the characteristic of ZnO in diarrhea prevention has drawn a great attention. Post-weaning diarrhea is one of the most common causes of morbidity and mortality for weaning piglets, resulting in reduced growth performance in piglets [13]. During the first two weeks after weaning, the gastrointestinal tract of piglets undergoes a dynamic stress process, likely due to nutritional, psychological, environmental and physiological factors [14]. In 1980s, it was found that feeding with pharmacological concentrations of ZnO (2000 to 4000 mg/kg) can reduce diarrhea and increase growth rates in weanling piglets [15]. As the antimicrobial growth promoters being banned in many countries, dietary high dose of ZnO have drawn a great concerns from researchers and farm workers [16–18].

Recently, some disadvantages have been found when high dose of ZnO were extensively uses in pig production. Bednorz et al. [19] found that feeding with ZnO at the dose of 2500 ppm increases the proportion of multi-resistant Escherichia coli in ileum and colon digesta. It provokes the question about the reasonability of high-level Zn supplementation as a result of the ban of antimicrobial growth promoters. On the other side, the bioavailability of Zn from ZnO is lower than that from other Zn sources, such as ZnSO_4_, Zn-methionine and Zn-lysine [20]. Feeding piglets with high dose of ZnO results in plenty of un-absorbable Zn being released to environment, finally increasing the risks of multiple dug resistance [19] and heavy metal contamination [21]. Thus, some researchers attempted to reduce the usage of ZnO in pig feed and enhance the biological effect of ZnO (especially on diarrhea). At the present time, changing regular ZnO powder into porous particles or nanoparticles is a common practice [22–24]. Morales et al. [23] found that low dose of porous ZnO (HiZox, 150 ppm) significantly improved the growth performance and health status of piglets compared to pharmacological dose of regular ZnO in the stater phase. On the other side, Cho et al. [25] found that compared with TiO_2_ nanoparticles, ZnO nanoparticles had higher absorption and more extensive organ distribution when administered orally in rat, suggesting it is a potential alternative form of regular ZnO powder. Thus, both porous ZnO and nano ZnO were deemed as potential candidates for replacing high-dose dietary regular ZnO in weaning piglets. Further evaluation of the tissue distribution, diarrhea prevention, anti-inflammatory effect of these ZnO products following oral administration in piglets is still needed to provide valuable information for assessing the merits of these particles. Therefore, we compared the effects of porous and nano ZnO on growth performance, diarrhea, tissue Zn distribution, blood biochemistry parameters, intestinal morphology and inflammation in weaning piglets. The possibility of replacing high-dose dietary regular ZnO with low-dose porous and nano ZnO was also assessed in this study.

## Materials and methods

### Animals and experimental design

This study was conducted according to the guidelines of the Declaration of Helsinki and all procedures involving animal subjects were approved by the animal welfare committee of the Institute of Subtropical Agriculture, Chinese Academy of Science.

A total of 128 piglets (Duroc× Landrace × Yorkshire, weaned at 35 ± 1 d with BW of 10.96 ± 1.25 kg) were used in a 28-d experiment. They were randomly allotted to four dietary treatments. Dietary treatments were replicated using eight pens (four piglets per pen, two males and two females). The experimental diets were formulated using corn and soybean meal supplemented with three forms of ZnO: regular powder, porous particles and nanoparticles. Porous ZnO (HiZox) was kindly offered by Animine Co., Ltd (Sillingy, France). Nano ZnO was purchased from Aoge Biotechnology Co., Ltd (Shanghai, China). The morphology of particles were measured using scanning electron microscopy (TM 1000, Hitachi Science Systems, Ltd., Japan) and Zetasizer Nano ZS (Malvern Instruments, Malvern, UK), and the data was showed in Supporting Information. The basal diet was used as negative control (NC) diet. The basal diet supplemented with standard commercial feed-grade ZnO (powder) at the dose of 3000 mg/kg (containing 2400 mg/kg Zn) was used as positive control (PC). Other two experimental diets were basal diet supplemented with 500 mg/kg porous ZnO (HiZ, containing 400 mg/kg Zn) and 500 mg/kg nano ZnO (ZNP, containing 400 mg/kg Zn), separately. All the diets were formulated to be iso-energetic and iso-nitrogenous and to meet the NRC (2012) nutrient requirements. All of the ZnO were pre-added in the Premix to allow they were at certain concentrations in different diets. The nutrient composition of basal diet is shown in Table 1. Piglets were allowed to have a 5-d adaption period before the trial begins. During the experimental period, piglets had free access to feed and drinking water at all times. Feed consumption from each pen was determined daily throughout the experimental period. Initial (day 0) and final (day 28) body weights were measured after 12 h fasting. Response variables measured included average daily feed intake (ADFI), average daily gain (ADG) and feed/gain ratio. The status of anal soft fecal contamination and swelling in each pen was examined and recorded every morning and afternoon during the experimental period. These data were then used to calculate the incidence rate of diarrhea according to the following formula:

The incidence rate of diarrhea=[total number of diarrhea piglets/(total number of piglets×days of experiment )]×100%

**Table 1:** Composition of basal diet (as-fed basis)^1^.

Ingredient	Content, %	Nutrient level	Content, %
Corn	58.00	DE (MJ/kg)	14.01
Soybean meal	17.00	CP	22.84
Extruded soybean	7.00	Ca	0.86
Fish meal	4.50	TP	0.75
Whey powder	4.50	Lys	1.13
Wheat bran	5.00		
Dicalcium phosphate	1.50		
Salt	0.25		
Limestone	1.10		
Lys	0.15		
Premix^2^	1		
Total	100		

^1^The dietary treatments were: negative control (NC), basal diet; positive control (PC), basal diet + 3000ppm ZnO; low HiZox (LHZ), basal diet + 200 ppm HiZox; high HiZox (HHZ), basal diet + 500 ppm HiZox; ZnO nanoparticles (ZNP), basal diet + 500 ppm ZnO nanoparticles

^2^Premix supplied per kilogram of diet: VA_1_, 500 IU; VD_3_, 200 IU; VE, 10 IU; VK_3_, 0.5 mg; VB_2_, 3.6 mg; VB_1_, 1.0 mg; VB_6_, 1.5 mg; biotin, 0.05 mg; folic acid, 0.3 mg; d-pantothen, 10 mg; nicotinic acid, 10 mg; choline, 500 mg.

### Sample collection

On day 28, 32 piglets (one piglet per pen) were randomly selected, and then killed according to our previous report [26]. Blood were sampled from a jugular vein, followed by the centrifugation at 3000g for 10 min at 4°C and stored at -80°C until analysis. Segments of longissimus dorsi muscle, liver, duodenum, jejunum and ileum were taken quickly. One part of the gut samples was kept in 10% neutral buffered formalin for histomorphometry analysis, other tissue samples were immediately frozen in liquid nitrogen and stored at -80°C for subsequent analysis.

### Analysis of small intestinal morphology

The intestinal segments (20 mm) were embedded in paraffin. Sections (5 μm) were cut and stained. The stained sections were subsequently used to determine villus height (μm) and crypt depth (μm) according to previous study [27].

### Analysis of blood samples

The serum concentrations of malondialdeyhde (MDA) and enzyme activities of diamine oxidase (DAO) and glutathione peroxidase (GSH-Px) were measured using spectrophotometric kits in accordance with the manufacturer’s instructions (Nanjing Jiangcheng Biotechnology Institute, China).

### Analysis of trace metal elements in muscle and kidney

Tissue samples (5 g) were sliced and added into a mixture of perchloric acid and nitric acid (15mL, v:v = 1:4) overnight. Then the mixture were heated to 80°C for 2h, followed by 110°C for 1h, 150°C for 1h, and maintained at 220°C until dried. The ashed samples were suspended with 15 ml 1% nitric acid and filtered before analysis. The filtrated solution was then aspirated into inductively coupled plasma-optical emission spectroscopy (ICP-OES, Agilent, 720 ES). The concentrations of copper (Cu), zinc (Zn), ion (Fe), calcium (Ca), manganese (Mn), chromium (Cr) and magnesium (Mg) in muscle and kidney were determined.

### RNA extraction and gene expression analysis

Total mRNA from duodenum, jejunum and ileum were isolated using TRIzol Reagent (TaKaRa, Dalian, China) according to the manufacturer’s instruction. The reverse transcription was performed according to our previous study [26]. Primers for interleukin 1 and 6 (IL-1, IL-6), tumor necrosis factor α (TNF-α), interferon γ (IFN-γ), trefoil factor 3 (TFF3) and nuclear factor erythroid 2-related factor 2 (Nrf2) were list in Table 2. β-actin was used as a housekeeping gene to normalize the relative change of each mRNA. Real-time PCR was performed according to our previous studies [26].

**Table 2:** The primer sequences used in this study.

Gene^1^	Primer squence (5’-3’)	Size (bp)	References
IL-1	F: GCTAACTACGGTGACAACAA R: TCTTCATCGGCTTCTCCACT	196	[28]
IL-6	F: CCTGTCCACTGGGCACATAAC R: CAAGAAACAACCTGGCTCTGAAAC	253	[29]
TNF-α	F: CATCGCCGTCTCCTACCA R: CCCAGATTCAGCAAAGTCCA	199	[30]
IFN-γ	F: GAGCCAAATTGTCTCCTTCTAC R: CGAAGTCATTCAGTTTCCCAG	140	[31]
TFF3	F: AGTGTGCCGTCCCTGCCAAG R: GCAGCCCCGGTTGTTGCACT	80	[32]
Nrf2	F: GAAAGCCCAGTCTTCATTGC R: TTGGAACCGTGCTAGTCTCA	190	[33]
β-actin	F: CCAGGTCATCACCATCGG R: CCGTGTTGGCGTAGAGGT	158	[31]

^1^ IL-1, interleukin 1; IL-6, interleukin 6; TNF-α, tumor necrosis factor α; IFN-γ, interferon γ; TFF3, trefoil factor 3; Nrf2, nuclear factor erythroid 2-related factor 2.

### Statistical analysis

All data were analyzed by one-way analysis of variance (ANOVA) to test homogeneity of variances via Levene’s test and followed with Ducan’s multiple comparison test (SPSS18.0 software). Data is showed as the mean ± standard error of the mean. Values in the same row with different superscript are significant (*P* < 0.05), while values with the same superscript are not significantly different (*P* > 0.05).

## Results

### Growth performance and the incidence rate of diarrhea

The result of growth performance and the incidence rate of diarrhea were summarized in Table 3. From weanling to 28d post-weaning, piglets in PC and ZNP groups showed significantly higher ADG than NC group (*P* < 0.05). Piglets in HiZ group had similar diarrhea incidence compared with those in PC group (*P* > 0.05). Besides, HiZ group had less diarrhea compared with ZNP group (*P* > 0.05).

**Table 3:** Growth performance and the incidence rate of diarrhea.

Item^1^	NC	PC	HiZ	ZNP
ADG (g/d)	329.91±23.34^c^	420.09±7.57^a^	367.41±13.38^b^^c^	377.04±12.09^a^^b^
ADFI (g/d)	601.82±11.17^c^	699.93±23.89^a^	651.61±9.59^b^	675.77±5.57^a^^b^
F/G	1.91±0.08^a^	1.69±0.03^b^	1.80±0.05^a^^b^	1.87±0.02^a^
Diarrhea incidence	9.15±0.08^a^	4.91±0.10^c^	5.13±0.07^c^	5.51±0.10^b^

^1^ADFI, average daily feed intake; ADG, average daily gain; F/G, feed/gain ratio. Data were shown as the mean ± SEM, n = 8.

^abc^ Mean values within different letters were significantly different (P<0.05).

### Trace metal elements in muscle and kidney

Dietary supplementation with different forms of ZnO had little impact on the concentrations of trace metal elements in muscle (Table 4). Piglets fed with or without ZnO did not influence the concentrations of trace metal elements in kidney, except for Zn. Piglets in PC group had the highest Zn concentration in kidney, while other three groups had similar Zn level in kidney.

**Table 4:** Concentrations of trace metal elements in muscle and kidney^1^.

Organ	Metal	NC	PC	HiZ	ZNP
Muscle (μg/g)	Cr	0.13±0.01	0.11±0.00	0.11±0.01	0.13±0.02
	Mn	0.08±0.01	0.06±0.00	0.06±0.00	0.07±0.01
	Cu	0.31±0.01	0.27±0.02	0.29±0.01	0.29±0.02
	Zn	1.5±0.06	1.51±0.05	1.38±0.13	1.39±0.09
	Fe	1.62±0.08	1.18±0.04	1.23±0.10	1.33±0.12
	Ca	22.59±1.9	23.9±0.5	21.53±1.6	22.82±0.10
	Mg	24.67±0.59	24.58±0.87	21.76±2.00	23.5±0.10
Kidney (μg/g)	Cr	0.09±0.01	0.09±0.00	0.1±0.01	0.1±0.01
	Mn	0.29±0.02	0.26±0.01	0.28±0.01	0.27±0.01
	Cu	4.82±0.46	6.12±0.53	4.92±0.76	4.65±0.48
	Zn	8.17±1.06^a^	20.23±1.38^b^	5.85±0.42^a^	6.71±0.62^a^
	Fe	0.29±0.02	0.26±0.01	0.28±0.01	0.27±0.01
	Ca	21.84±0.61	25.54±2.48	19.69±1.07	21.32±2.09
	Mg	16.55±0.68	18.21±0.22	16.58±0.45	16.24±0.56

^1^ Data were shown as the mean ± SEM, n = 8

^ab^ Mean values within different letters were significantly different (P<0.05).

### DAO, GSH-Px and MDA in serum

**Fig 1:**
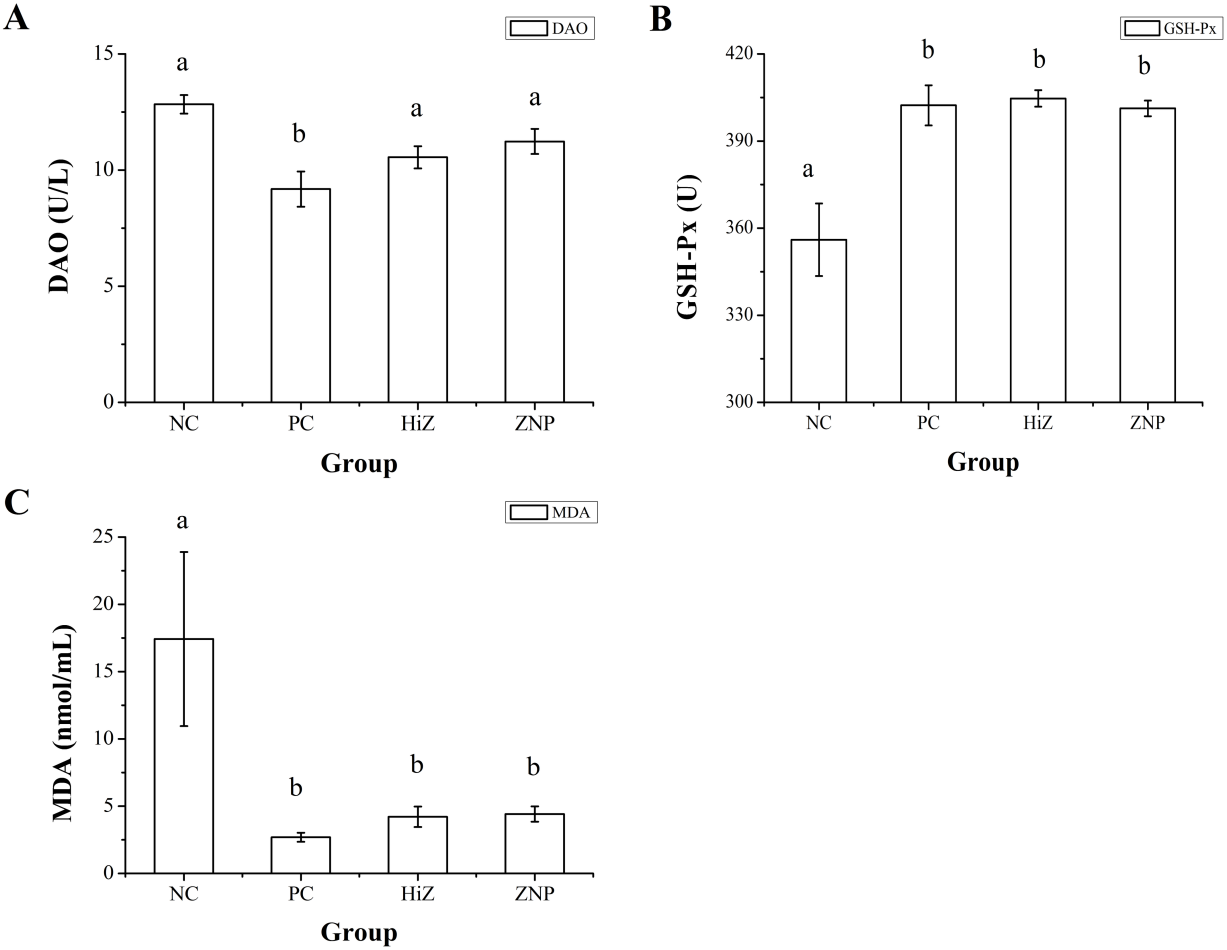
Activities of diamine oxidase (DAO) (A) and glutathione peroxidase (GSH-Px) (B), serum malondialdeyhde (MDA) concentration (C) in four groups. Data were shown as the mean ± SEM, n = 8. ^a,b^Mean values within different letters were significantly different (P<0.05).

MDA concentration and activities of DAO and GSH-Px in serum were presented in Fig 1. Serum DAO activities were significant higher in NC group than in other groups (*P* < 0.05). Compared with NC group, piglets fed with ZnO (PC, HiZ and ZNP groups) had higher GSH-Px activities and lower MDA concentration in serum (*P* < 0.05). Piglets fed with HiZ and ZNP diets had no significant differences in DAO, GSH-Px and MDA levels (*P* > 0.05).

### Intestinal morphology

As Table 5 showed that piglets in PC group had higher duodenal villus height and deeper jejunal crypt depth compared with other groups (*P* < 0.05). Piglets in PC, HiZ and ZNP group had deeper ileal crypt depth of than those in NC and LHZ group (*P* < 0.05).

**Table 5:** Effect of different sources of ZnO on intestinal morphology of weanling pigs^1^.

	Item (μm)	NC	PC	HiZ	ZNP
Duodenum	Villus height	386.60±8.73^b^	408.32±5.95^a^	376.73±2.42^b^	373.63±9.15^b^
	Crypt depth	154.49±10.60	156.30±6.45	153.68±8.46	151.05±10.47
	V/C^2^	2.50±0.18	2.61±0.15	2.49±0.14	2.41±0.15
Jejunum	Villus height	334.73±11.27	360.58±13.66	355.79±26.18	352.89±13.81
	Crypt depth	133.79±6.93^b^	156.7±3.91^a^	150.03±5.08^a^^b^	133.79±7.95^b^
	V/C	2.46±0.15	2.48±0.07	2.42±0.15	2.68±0.14
Ileum	Villus height	320.40±15.01	305.40±20.21	321.03±16.78	322.45±14.86
	Crypt depth	141.51±6.14^a^	120.47±1.66^b^	126.96±5.43^b^	119.73±4.07^b^
	V/C	2.30±0.11	2.59±0.16	2.43±0.13	2.50±0.20

^1^ Data were shown as the mean ± SEM, n = 8

^2^ V/C = Villus height/ Crypt depth

^a,b^ Mean values within different letters were significantly different (P<0.05).

### Intestinal inflammation

**Fig 2:**
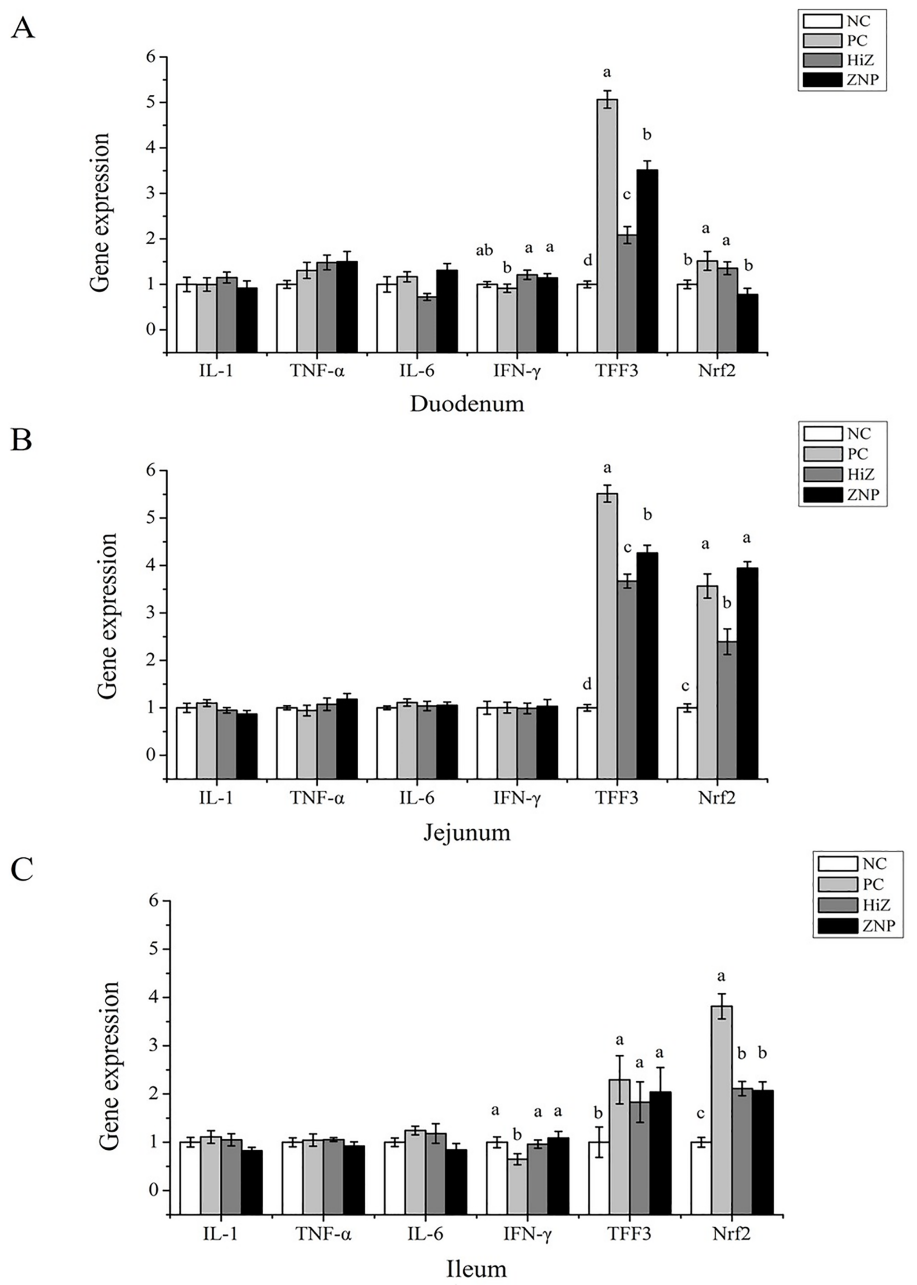
Intestinal relative mRNA levels of inflammatory cytokines. IL-1, interleukin 1; IL-6, interleukin 6; TNF-α, tumor necrosis factor α; IFN-γ, interferon γ; TFF3, trefoil factor 3; Nrf2, nuclear factor erythroid 2-related factor 2. Data were shown as the mean ± SEM, n = 8. ^a,b^Mean values within different letters were significantly different (P<0.05).

The result presented in Fig 2 showed that the dosage form of ZnO did not influence the mRNA expressions of IL-1, TNF-α and IL-6 in small intestine. PC diet down-regulated the mRNA level of IFN-γ in duodenum and ileum compared with HiZ and ZNP group (*P* < 0.05). Dietary supplementation with ZnO (PC, HiZ and ZNP group) up-regulated the mRNA levels of TFF3 and Nrf2 compared with NC group (*P* < 0.05). On the other side, in duodenum and ileum, the mRNA level of TFF3 is lower in HiZ group compared with ZNP group (*P* < 0.05). The duodenal mRNA level of Nrf2 is lower, while jejunal mRNA level of Nrf2 is higher in HiZ group compared with those in ZNP group (*P* < 0.05).

## Discussion

Zn is an important micronutrient for the overall health and development of infants and young children. It has multiple effects on pathophysiological processes, including the absorptive and secretory processes [34, 35], the gut associated immune process [36], and the metabolic activity of the intestinal microbiota [37, 38]. Besides, numerous animal and human studies demonstrated the benefits of Zn supplementation on inflammation and infectious diseases [39, 40]. Therefore, Zn and Zn compounds have been frequently utilized as antimicrobial agents and growth promoter. In pig production, dietary supplementation with high doses of ZnO (2000–3000 mg/kg) have been frequently used to improve performance and to reduce the infectious diarrhea in newly-weaned piglets because of its antibacterial activity [41]. The antibacterial activity of ZnO is considered to be due to the generation of hydrogen peroxide (H_2_O_2_) from its surface [42]. Therefore, it is assumed that increasing the surface area of ZnO particle (decreasing particle size) can elevate the efficient of the H_2_O_2_ generation and the antibacterial activity. Osamu Yamamoto confirmed the suspect and found that the antibacterial activity of ZnO increased with decreasing particle size and increasing powder concentration [43]. Here we used low levels of porosity particles and nanoparticles of ZnO (both of them possess large surface layer) to investigate their potential to replace high dose of ZnO in piglet diets. We found that both porous and nano ZnO (HiZ and ZNP group) improved the growth performance, and showed similar or even better effect on reducing diarrhea than high-dose of regular ZnO (PC group). Besides, porous ZnO (HiZ group) had better effect on reducing diarrhea than nano ZnO (ZNP group). These results suggested that porous and nano ZnO possess more effective surface area than regular ZnO did under the same concentration. It also implied that further reducing the dose of porous and nano ZnO maybe still in effect.

To exert anti-diarrhea effect, the dosage of dietary regular ZnO needs to be high enough (approximately 2000–3000 mg/kg in diet of weaning piglet). Even when porous and nano ZnO are used as substitute, the dosage of ZnO added in feed is still higher than that of other Zn supplements, such as ZnSO4 and amino acid-chelated Zn. On the other side, the bioavailability of dietary ZnO is relative lower than ZnSO_4_ and amino acid-chelated Zn. Even so, there would be still a large amount of Zn enter the body by absorption through intestine when piglets fed with high level (pharmacological level) of ZnO [20]. Once Zn is absorbed by intestine, it is predominantly bound to proteins in the circulation [44]. Under normal circumstances, urinary loss of Zn is very low, and large amount of reabsorbed Zn would be stored in kidney waiting for transportation [45]. Here we found that feeding with regular ZnO at 3000 mg/kg (PC group) results in a large accumulation of Zn in kidney, which is extremely higher than other groups. However, high Zn in kidney may contribute to kidney stone formation, a common urinary condition that can cause excruciating pain [46]. Therefore, replacing high-dose dietary ZnO with low-dose porous and nano ZnO, in order to reduce Zn accumulation in kidney, is beneficial to the health of urinary system.

Zn is an important anti-stress factor. It is a fundamental element of more than 200 metalloenzymes, including many antioxidant enzymes, and affects activity and stability of many of them [47]. On the other side, weaning stress is often associated with oxidative stress and presented as the lipid peroxidation, elevated generation of MDA and reduced the activity of antioxidant enzymes [26]. And Zn plays an antioxidative role during weaning period in piglets. Thus, it is important to investigate the activities of antioxidant enzymes in weaning piglets when the usage of Zn is reduced. In this study, we found that dietary ZnO (PC, HiZ and ZNP group) significantly decreased the MDA level in serum compared with NC group, suggesting that both high dose of regular ZnO and low dose of porous and nano ZnO are able to reduce lipid peroxidation effectively. On the other side, ZnO has been reported to affect expression of proteins related to glutathione metabolism and favorably increased the expression of antioxidative proteins [48]. Elevation of GSH-Px activities may be an adaptive mechanism secondary to the increase of oxidative stress [49]. Consistent with this notion, we found that GSH-Px activity was significantly increased in PC, HiZ and ZNP group, implying that dietary supplementation with porous and nano ZnO at low concentration can effectively promote adaption to the oxidative stress as high level of regular ZnO did. DAO is an intracellular enzyme and widely distributed in intestinal villous of mammalians. Its activity is especially high in the jejunum and ileum [50]. An increase amount of DAO released into blood is considered as a signal of damage in intestinal mucosal integrity [51, 52]. Considering that Zn plays an important role in maintain epithelial barrier integrity and function [8, 53], we tested DAO activity in serum and found that piglets fed with basal diet (NC group) showed a higher DAO activity than other three groups, suggesting that low level of porous and nano ZnO, as well as high level of regular ZnO are beneficial in maintaining intestinal mucosal integrity. To get a better insight into the effect of different dosage forms of ZnO on the intestinal structure, we determined the intestinal villus height and crypt depth in piglets. A decrease in ileal crypt depth was observed in PC, HiZ and ZNP group, suggesting an effective protection against intestinal injury [27].

The weaning process is associated not only with intestinal integrity, but also with the intestinal inflammation. It has been well documented that weaning triggers the up-regulation of pro-inflammatory cytokines in the intestine, such as TNF-α, IL-6, IL-1β and INF-γ [54–56]. The inflammatory response and overproduction of pro-inflammatory cytokines result intestinal barrier dysfunction [54, 57]. In some studies, mRNA levels of TNF-α, IL-6 and IL-1 decreased with increasing concentration of dietary Zn in pigs [58, 59]. However, in the present study, dietary addition of ZnO (PC, HiZ and ZNP group) did not affect the mRNA expressions of IL-1, TNF-α and IL-6 after a 28-d feeding process. It has been demonstrated that weaning is associated with a transient up-regulation of inflammatory cytokine mRNA content on days 3 to 4 post weaning, and most of them rapidly return to pre-weaning values after day 9 post weaning [56, 58]. This might be why mRNA levels of IL-1, TNF-α and IL-6 were not affected by the dietary treatment in the present study. TFF3 and Nrf2 are important inflammatory cytokines. They have a major impact on maintenance of healthy mucosal surfaces [60, 61]. In the intestine, weaning could result in an increase of TFF3 mRNA levels, which is thought to be beneficial for the epithelial repair [62]. And increase of Nrf2 expression improves the expression of antioxidant genes and inhibits the expression of pro-inflammatory cytokines [63]. In this study, TFF3 and Nrf2 were up-regulated in the ZnO-fed group. Interestingly, ZNP supplementation had better effects on up-regulating TFF3 and Nrf2 levels in duodenum and jejunum than HiZ did, implying that nano ZnO may possess higher anti-inflammatory capacity than porous ZnO.

## Conclusions

Dietary supplementation with low dose of porous and nano ZnO has similar (even better) effect on improving growth performance and intestinal morphology, reducing diarrhea and intestinal inflammatory as high dose of regular ZnO in weaning piglets. Compared with nano ZnO, porous ZnO has better effect on reducing diarrhea. But nano ZnO shows better effect on un-regulation of intestinal TFF3 and Nrf2 levels, suggesting it may possess higher anti-inflammatory capacity than porous ZnO. Overall, both porous and nano ZnO can be used as alternatives to high dose of regular ZnO in weaning piglets.

## Supporting information

S1 Fig TEM images of (a) porous ZnO and (b) nano ZnO. S1 Fig shows the morphology of the porous ZnO and nano ZnO through TEM. S1 Fig A shows that porous ZnO have a rough surface and exhibit a spongelike structure. S1 Fig B shows that nano ZnO are spherical in shape with a uniform size, and are found as aggregated particles.
(TIF)

S2 Fig Particle size distribution of porous ZnO and nano ZnO. S2 Fig A shows that approximately 80% of the porous ZnO particles have a size ranging from 0.1 mm to 0.2 mm. S2Fig B shows that nano ZnO have much smaller particle size (most of them are less than 0.1 μm) than porous ZnO.
(TIF)
