# Correction: Comparison of porous and nano zinc oxide for replacing high-dose dietary regular zinc oxide in weaning piglets

**DOI:** 10.1371/journal.pone.0188587

**Published:** 2017-11-17

**Authors:** Lina Long, Jiashun Chen, Yonggang Zhang, Xiao Liang, Hengjia Ni, Bin Zhang, Yulong Yin

There is an error in Table 1: the nutrient level for CP is 17.98, not 22.84.

The incorrect table was used for Table 3. The published data of ADG, ADFI and F/G from weanling to 28d post-weaning are the ADG, ADFI and F/G from weanling to 14d post-weaning. The data of diarrhea incidence is correct. Please see the correct Table 3 here.

**Table 3:** Growth performance and the incidence rate of diarrhea.

Item^1^	NC	PC	HiZ	ZNP
ADG (g/d)	438.62±6.31^b^	529.24±7.71^a^	500.22±6.22^b^	520.41±2.78^a^
ADFI (g/d)	870.08±10.19^c^	1001.57±12.09^a^	978.06±2.82^a^^b^	962.83±5.66^b^
F/G	1.99±0.01^a^	1.90±0.02^b^	1.94±0.02^b^	1.83±0.01^c^
Diarrhea incidence	9.15±0.08^a^	4.91±0.10^c^	5.13±0.07^c^	5.51±0.10^b^

^1^ADFI, average daily feed intake; ADG, average daily gain; F/G, feed/gain ratio. Data were shown as the mean ± SEM, n = 8.

^abc^ Mean values within different letters were significantly different (P<0.05).
