# Supplementary figures and images for: Comparison of porous and nano zinc oxide for replacing high-dose dietary regular zinc oxide in weaning piglets
Source: PLoS One. 2017 Aug 8;12(8):e0182550. doi: 10.1371/journal.pone.0182550 (PMC5549748; doi:10.1371/journal.pone.0182550)

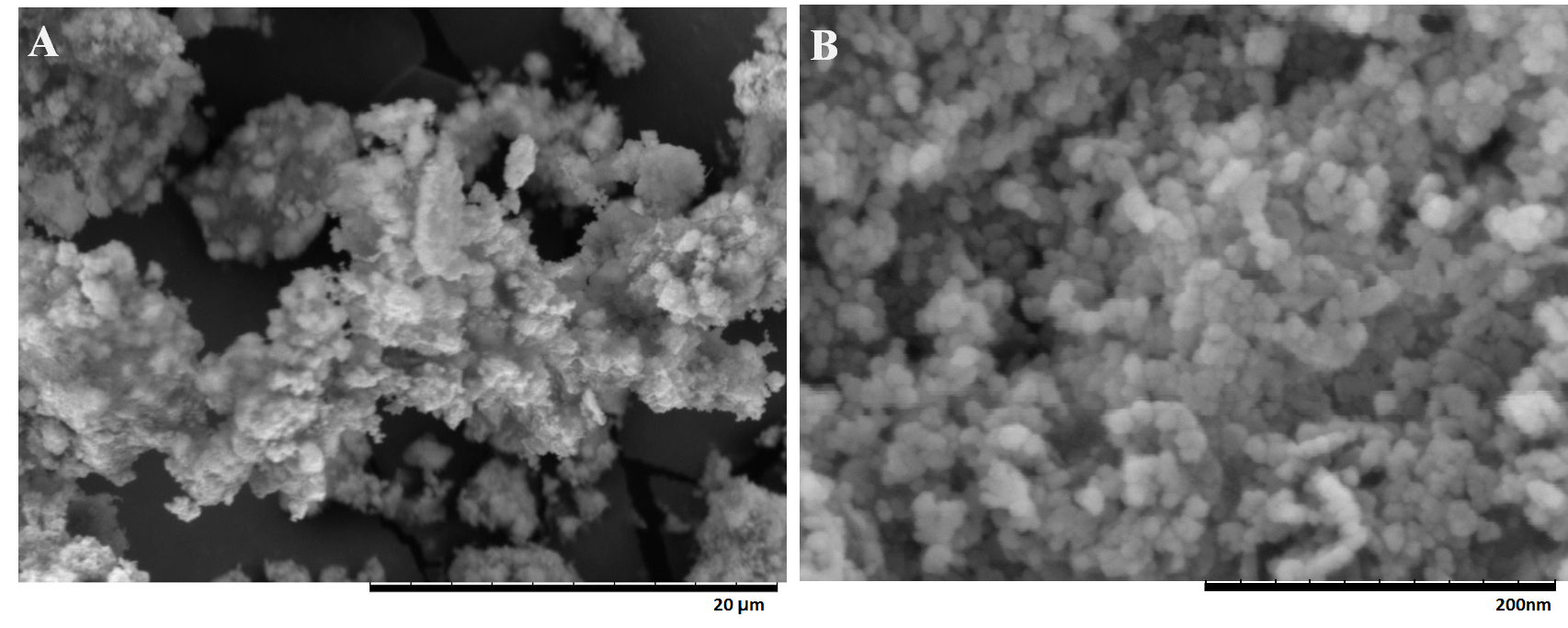

Supplement: S1 Fig — shows the morphology of the porous ZnO and nano ZnO through TEM. S1 Fig A shows that porous ZnO have a rough surface and exhibit a spongelike structure. S1 Fig B shows that nano ZnO are spherical in shape with a uniform size, and are found as aggregated particles. (TIF) [file pone.0182550.s001.tif]

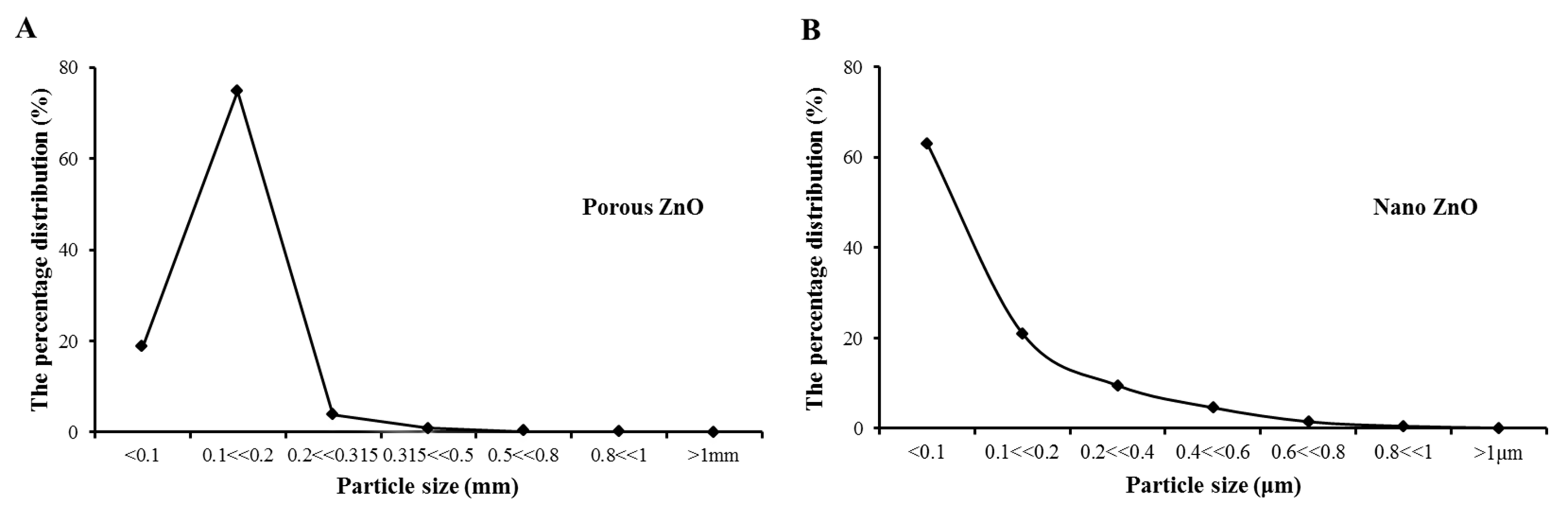

Supplement: S2 Fig — A shows that approximately 80% of the porous ZnO particles have a size ranging from 0.1 mm to 0.2 mm. S2Fig B shows that nano ZnO have much smaller particle size (most of them are less than 0.1 μm) than porous ZnO. (TIF) [file pone.0182550.s002.tif]
